# A nurse‐led intervention study: Promoting compliance with Diskus Inhaler use in asthma patients

**DOI:** 10.1002/nop2.10

**Published:** 2014-11-28

**Authors:** Elaine Mac Hale, Richard W. Costello, Seamus Cowman

**Affiliations:** ^1^ Clinical Research CentreRoyal College of Surgeons in IrelandDublinIreland; ^2^ Department of Medicine Respiratory Research DivisionRoyal College of Surgeons in IrelandDublinIreland; ^3^ School of Nursing & MidwiferyRoyal College of Surgeons in IrelandAdliyaBahrain

**Keywords:** Adherence, asthma, compliance, Diskus, education, inhaler technique, nurse, nursing

## Abstract

**Aim:**

To examine the impact of a nurse‐led patient assessment and education programme in promoting compliance with inhaler use in asthma patients.

**Design:**

A quasi‐experimental pre‐test and post‐test design.

**Methods:**

A sample of asthmatic patients (*N *=* *21) were recruited from the population of patients attending an asthma clinic. An Inhaler Proficiency Schedule (IPS) was developed and validated. At each visit, participants were requested to demonstrate their inhaler technique. The participants were investigated as to their confidence level with self‐administration of their inhaler and adherence to prescribed doses. This information was recorded on a Patient‐Reported Behaviour (PRB) questionnaire.

**Results:**

Technique, compliance and patient confidence levels improved with nurse‐led education repeated over three visits; this was sustained on measurement at 6 months following completion of the education programme.

## Introduction

There are approximately 470,000 individuals with a diagnosis of asthma in Ireland; this ranks Ireland as the country with the fourth highest prevalence per head of population in the world (Asthma Insights and Reality in Europe (AIRI) 2005). Inhalation medication is an essential therapy for patients with asthma. Inadequate patient instruction and poor patient inhaler technique have a significant impact on asthma control. Effective treatment and disease management requires adherence to inhalation therapies; poor adherence and technique pose a challenge to nurses, clinicians and patients. Despite the prevalence of asthma and the presence of the Global Initiative for Asthma (GINA) guidelines for the management of asthma, inhaler technique remains poor.

This paper reports on a study that examined the benefits of a nurse‐led programme to educate asthma patients on correct inhaler technique, specifically a Diskus inhaler. The study presents the impact of the education on patient compliance and adherence with inhaler use.

## Background

Incorrect inhaler technique is associated with poor asthma control (Crompton *et al*. 2006). Patients may receive treatment, but without proper education and training in inhaler technique, the therapeutic benefit is below optimal (Lavorini *et al*. 2008). Clinical benefits depend on the patient's ability to use the device correctly and adherence to prescribed frequency. Real‐life observational studies that have evaluated patient inhalation techniques have shown frequent improper use of inhalers (Webb *et al*. 2001, Restrepo & Gardner 2010).

Management of Asthma becomes suboptimal due to poor adherence to evidence‐based guidelines and underdiagnosis (Ramsey 2000). Patient benefit and therapeutic outcomes depend on the ability of the patient to use the device and on their adherence to the dosing regimen (Dolovich *et al*. 2005, Lavorini *et al*. 2008, Restrepo *et al*. 2008).

There is evidence suggesting that patient errors in the use of inhaler devices are common; many inhalers are difficult to use. (Van Beerendonk *et al*. 1998). Although there have been numerous studies on education in inhaler technique for both the educator and the patient, it has been demonstrated that patients are consistently using incorrect technique and this has negative outcomes on their medical condition.

Patients are reporting that they have not been taught how to use their inhaler by anyone, least of all a healthcare provider. Sestini *et al*. (2006), reported that primary care physicians are not familiar with relevant features of currently available inhalers. It has been shown that adherence is poor in patients with asthma (Takemura *et al*. 2010). Non‐adherence and non‐compliance to treatment regimens are a constant challenge to nurses and other health professionals. Compliance or adherence to prescribed treatment is not easily attained and is affected by a multitude of issues relating to the patient, the doctor and the nurse.

Simple instructions in asthma self‐management improve adherence with inhaler devices, but despite this, improvements can decrease over time – even with return clinic visits (Rau 2005, Lasmar *et al*. 2009). The problem of adherence is compounded when more than one inhaler is required. For example, when two medications are prescribed, approximately 50% of patients are adherent to only one (Corden *et al*. 1997, Schlenk *et al*. 2004).

The National Collaborating Centre for Primary Care (NCCPC) (2009) states that while there are many causes of non‐adherence, they can be summarized into two overlapping categories: intentional and unintentional non‐adherence:

- *Intentional non‐adherence* is when the patient decides not to follow the recommendations. Refusing to take medication for fear of adverse effects constitutes intentional non‐adherence (Rau 2005).
- *Unintentional non‐adherence* occurs when the patients agree to the treatment, but are prevented from doing so by reasons beyond their control. Examples include poor comprehension and/or poor recall of instructions, a difficulty in administration of their treatment, or simply forgetting to take their medication.

Health promotion and primary prevention of disease by nurses has evolved into an essential and critical aspect of their role and is practised across many therapeutic areas (Benson & Latter 1998, Cutler 1999).

## The study

The aim of this study was to examine the impact of a nurse‐led education programme in promoting compliance with inhaler use in asthma patients.

### Design

A single‐centre, quasi‐experimental, pre‐test and post‐test design was used. The study was conducted in the respiratory clinic of an Irish teaching hospital. Convenience sampling was applied to select patients. Despite the limited time window and resources available for the study, the researcher used the largest sample available. As there was also a risk of bias with non‐probability convenience sampling, the researcher ensured homogeneity of the sample by using a set of inclusion and exclusion criteria to identify suitable participants. The inclusion criteria for entry into the study included patients:

- With a clinical diagnosis of asthma,
- Willing to provide voluntary informed consent,
- Age 18 years or older at time of consent,
- Able and willing to take inhaled asthma medication.

The sample included ten men (*n *=* *10) with a mean age of 42·3 (sd 13·5) years and eleven women (*n *=* *11) with a mean age of 39·7 (sd 15·5) years.

### Method

Through the use of a newly developed and validated Inhaler Proficiency Schedule (IPS) (Mac Hale & Cowman 2012a), (Appendix 1), participants were assessed for competence with inhaler use. The elements of the IPS included ten steps in best practice in using the Diskus device. A nurse‐led intervention education programme was then implemented. Measurements with the IPS were recorded at baseline (month 1), month 3 and finally month 6 after the education intervention (the follow‐up visit), to examine the sustainability of the change. The researchers also used a questionnaire to record participants’ behaviour in relation to their inhaler use on a Patient Reported Behaviour questionnaire (PRB) (Mac Hale & Cowman 2012a, b), (Appendix 2). The PRB to be completed by participants comprised a series of specific questions in relation to their condition, confidence level with self‐administration of their inhaler and adherence to prescribed frequency of use.

The IPS and the PRB were adapted and developed by the researchers and were based on a comprehensive literature search, clinical experience and observation. For the purpose of this study and in consideration of the patient cohort, as respondents, the researchers concentrated on content and face validity. Face validity refers to the ‘obviousness’ of a test, i.e. the degree to which the purpose of the test is apparent to those taking it. (Bornstein 2004). Tests wherein the purpose is clear, even to naïve respondents, are said to have high face validity (Nevo 1985).

The IPS developed was validated by the Professor/Consultant in Respiratory Medicine and Clinical Nurse Specialist in Respiratory Nursing. The PRB Questionnaire was adapted from questionnaires used in previous studies and was validated by the Professor/Consultant in Respiratory Medicine and Clinical Nurse Specialist in Respiratory Nursing.

At each of the three study visits, the participants were asked to demonstrate how they used their inhaler. This was measured and recorded against the IPS checklist. This tool allowed the researcher to record specific observations made the participant demonstrated the use of his/her inhaler. Any errors in technique were identified on the IPS and corrective education was provided individually to the respondent, by the researchers, as required.

Inhaler technique data and patient reported behaviour data were collected (in the period January 2011–January 2012) at baseline, month 3 and month 6 by the same research nurse using the IPS and the PRB tools. The PRB was completed by each participant at each visit. Additional data collected at baseline included age, gender, diagnosis, previous Diskus inhaler use, previous inhaler technique instruction and whether the patient had an exacerbation in the past year (an exacerbation in this study having been defined as the worsening of respiratory symptoms requiring either steroid and/or antibiotic use, or one hospitalization for asthma).

### Nurse‐led intervention

A nurse‐led intervention included the provision of an education programme to study participants. The intervention protocol included the following steps:

- Competency assessment – On visit 1, participants were asked to demonstrate the use of their inhaler and they were assessed using the ten‐step IPS instrument. The results were documented;
- The completion by the participant of the PRB provided additional important information on the participant's self‐declared abilities, competency and compliance;
- A nursing consultation was initiated and each participant was provided with feedback on the use of their inhaler;
- In the event of one step, or several steps, of the IPS not being completed satisfactorily, the participant was provided with training including a demonstration and check on his/her ability to correct the deficient step(s) of the IPS;
- Related and appropriate education on asthma and the use of medication was provided during the consultation;

The above sequence of events was maintained in subsequent visits.

Statistical Analysis was performed using PASW (Predictive Analytics Software) Version 18. The Independent Samples *t*‐test was used to analyse the IPS data for two patient groups – those with previous experience using the Diskus inhaler and those who had no prior experience of using the Diskus inhaler (*P* ≤ 0·05).

Research Ethics approval was sought and obtained from the Hospital Ethics Committee where the study was conducted. A Patient Information Leaflet (PIL) was developed and given to each participant prior to obtaining written consent. Participants were assured of confidentiality and were informed of the option to withdraw at any time. Following the informed consent process where consent was freely given, these participants were recruited to this study.

## Results

Twenty‐one participants aged between 20–67 years, with a mean age of 40·95 years, were enrolled in the study. The sample included 10 men, with a mean age of 42·3 (sd 13·5) years, and 11 women with a mean age of 39·7 (sd 15·5) years. Tests for normality indicated that the distribution of participant population ages was within normal range (Kolmogorov–Smirnov test, *P* > 0·05).

The participants had between 1–40 years of asthma diagnosis with mean years of asthma diagnosis of 17·3 (sd 12·4) years. The mean years of asthma diagnosis for male participants (*n *=* *10) was 14·6 (sd 12·7) years. Female participants (*n *=* *11) had a mean years of asthma diagnosis of 19·7 (sd 12·3) years.

Based on the twenty‐one participants enrolled in the study, three participants did not complete all visits, while one participant had his/her medication/inhaler changed prior to the follow‐up visit. Therefore, statistical analysis of the IPS data was based on a core group of 17 patients who completed all stages of data collection. From the 17 participants, 41% (*n *=* *7) had been prescribed, and were using, a Diskus inhaler, while 59% (*n *=* *10) had not used a Diskus inhaler previously.

It was identified that 81% of participants (*n *=* *17) had at least one asthma exacerbation in the year prior to entering the study and the number of reported exacerbations ranged from 1–10 incidents. Males (70%) reported at least one exacerbation, with a range from 1–6 incidents and 91% of female participants reported at least one exacerbation, with a range from 1–10 incidents. At the follow‐up data collection point (month 6), participants were asked if they had visited their GP for their asthma in the previous 3 months. Five participants (*n *=* *5) had visited their GP. The number of visits by these participants to their GP ranged from 1–3.

### Inhaler Proficiency Schedule (IPS) results

**Figure 1:**
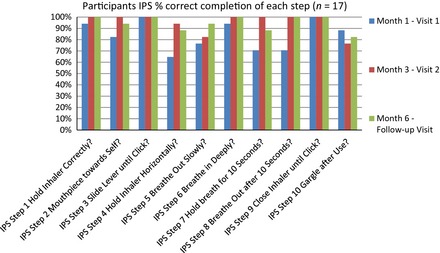
Participant IPS% correct completion of each step (*n *=* *17).

The correct Diskus completion rates, by participants, for each step of the IPS checklist are outlined in Figure 1. The results indicate a trend of improvement in participants' performance from month 1 to month 3, following completion of nurse‐led education, a pattern that is sustained to month 6. The total mean scores per participant for all ten steps of the IPS, as completed for month 1, month 3 and month 6, the follow‐up visit, are outlined in Table 1. As each step scores 0 for incorrect performance and 1 for correct performance, the maximum possible score per completed IPS per participant per visit is 10. The data indicate that mean participant scores improved from month 1 ‐ month 3 and were maintained in month 6.

**Table 1:** Participant (*n *=* *17) IPS Scores: month 1, month 3 and month 6.

Visit reference	*N*	Minimum	Maximum	Mean	SD
Month 1 – Visit 1	17	4·00	10·00	8·4118	2·12305
Month 3 – Visit 2	17	8·00	10·00	9·5294	0·62426
Month 6 – Follow‐up Visit	17	7·00	10·00	9·4706	0·87447

Participants were divided into two groups: those with previous Diskus experience and those with no previous Diskus experience. A secondary analysis of the data identified an even greater improvement in performance for the ‘previous Diskus use’ group on subsequent visits when compared with their visit 1 results. The data show that while the previous Diskus experience group performed poorly on visit 1, the benefit of education dramatically increased their performance at visit 2 and this improvement in performance was largely maintained at the 6‐month follow‐up visit. Participants new to Diskus score well after training on visit 1 and maintain their performance over 6 months. By visit 2, both groups are performing similarly and this is maintained throughout the data collection period (Table 2). Whilst the performance of the two groups is statistically significantly different at month 1, the impact of training and the improvement in scores eliminate the statistical difference between the two groups at month 3 and month 6 (Table 2).

**Table 2:** IPS Scores for participants with no Diskus experience (n = 10) and participants with Previous Diskus experience (n = 7)

Visit reference	Previous Diskus use?	*N*	Mean	SD	*t*	*P* ≤ 0·05
Month 1 – Visit 1	No Previous Diskus use	10	9·8000	0·63246	5·26	0·000
	Previous Diskus use	7	6·4286	1·90238		
Month 2 – Visit 2	No Previous Diskus use	10	9·6000	0·51640	0·54	0·594
	Previous Diskus use	7	9·4286	0·78680		
Month 6 – Follow‐up Visit	No Previous Diskus use	10	9·5000	0·70711	0·16	0·875
	Previous Diskus use	7	9·4286	1·13389		

### Patient Reported Behaviour (PRB)

On visit 2, there was an improvement in patient reported behaviour and it is noted that 94% (*n *=* *16) of participants took their inhaler at the correct time as compared with 65% (*n *=* *11) on visit 1; 88% (*n *=* *15) of participants now believed their inhaler to be effective as compared with 59% (*n *=* *10) on visit 1. Whereas 65% (*n *=* *11) of participants reported being careless about using their inhaler some of the time or all of the time as per visit 1, this trend was reversed with 65% (*n *=* *11) of participants responding that they were never careless about using their inhaler. Although participants did not remember to take their inhaler, this was reduced from 71% (*n *=* *12) on visit 1 to 65% (*n *=* *11) on visit 2. Only 6% (*n *=* *1) of participants either stopped using their inhaler or used it less than prescribed because they felt better compared with 41% (*n *=* *7) on visit 1.

At month 6 follow‐up visit, patient improvements with Diskus use were maintained. It is important to note that 6 months after the intervention, 88% (*n *=* *15) of participants still took their inhaler at the correct time; 88% (*n *=* *15) still believed their inhaler was effective. Notably 71% (*n *=* *12) of participants were never careless about using their inhaler. Compliance with Diskus use improved and participants who never forgot to take their inhaler increased from 29% (*n *=* *5) on visit 1, to 35% (*n *=* *6) on visit 2 and 53% (*n *=* *9) on the follow‐up visit.

On entering the study, 18% (*n *=* *3) had never been shown how to use their inhaler by a pharmacist. Notably, only 12% (*n *=* *2) reported having any concerns about taking their inhaler. It is important to note that 35% (*n *=* *6) reported that they had not taken their inhaler on the day of visit 1. Various explanations were offered, such as ‘forgot’, ‘was rushing’ and most worryingly, the fact that the canister was empty.

**Figure 2:**
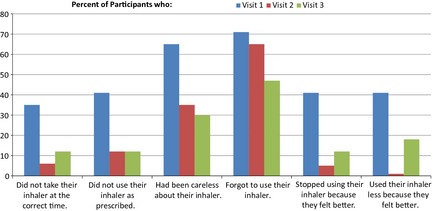
Overview of participant's responses to questions, to measure non‐compliance, both intentional and un‐intentional.

During the course of the study, participants reported increased compliance and confidence in: using their inhaler correctly and as prescribed; in the effectiveness of their inhaler: and in being compliant with inhaler use. Figure 2 summarizes the participant responses to questions to measure non‐compliance, both intentional and unintentional. The data were gathered across three visits and the trends show significant improvements in compliance levels from the first visit.

## Discussion

The purpose of this study was to evaluate the effectiveness of a nurse‐led education programme to improve inhaler technique and promote compliance with inhaler use in patients with asthma. A total of 17 participants who attended all three data collection points were included in the study.

Through the use of a ten‐step Inhaler Proficiency Schedule, it is notable that on initial assessment, only two steps were correctly completed by all participants and these were linked to the opening and closing of the inhaler device. No one critical step was successfully completed by all participants at the first visit. This was an unexpected finding, particularly given the mean age of asthma diagnosis for participants was 17.3 years.

Participants who had previous use of Diskus inhalers performed poorly on their initial visit. This concurs with the literature, which shows that experience of a device does not automatically guarantee good technique (Lavorini *et al*. 2008). The data also show that once educated in inhaler use, the IPS scores of the prior‐experience Diskus users improved to similar levels as the new‐to‐experience Diskus group. It is important to note that education was retained during the 3‐month gap between training at visit 1 and 2 and the follow‐up third visit, with participants demonstrating sustained competence with inhaler technique. This is consistent with study findings of Giraud *et al*. (2011), that education intervention results in good inhaler technique. However, the data also show that participants are capable of performing some steps incorrectly, even though they performed them correctly on a previous assessment. This is also in keeping with the literature, which reports that immediately after face‐to‐face instruction, participants are sometimes observed making mistakes in the use of their inhaler (Brocklebank & Ram 2001).

The four most common errors identified among study participants in using the Inhaler Proficiency Schedule were: incorrect inhaler positioning, no exhalation before breathing in, no breath hold and no slow exhalation after breath hold. These results are reflected in the published literature (Lavorini *et al*. 2008). In this study, some participants expressed pleasure that errors were identified in their technique as they felt that correction of same would lead to an improvement in their condition and quality of life.

The Patient Reported Behaviour (PRB) questionnaire was designed to capture intentional and unintentional non‐adherence and was completed at each visit. All questions relating to non‐adherence improved as the study progressed and again this is reflected in the literature (Takemura *et al*. 2010).

Three questions of the PRB related to participant confidence in using their inhaler correctly, taking it at the right time and using the prescribed number of puffs and the effectiveness of their inhaler. The results at visit 1 showed that 88% of participants expressed no concern or difficulties with using their inhaler, while 64% of the participants believed they were using their inhaler correctly. However, the IPS data from visit 1 showed that only 14% of participants actually took their inhaler correctly. However, following inhaler technique education, the participants became aware that they had scored themselves incorrectly and also expressed surprise that they had been taking their inhaler incorrectly; the participants were more receptive to education as a result. This suggests that there is a large theory–practice gap that needs to be narrowed and ideally eliminated.

Three PRB questions also focused on compliance, including participant carelessness and forgetfulness in use of their inhaler and ceasing to use their inhaler. The study shows increasing levels of compliance over time. This could also be because the participants gained more insight into the benefits of a ‘preventer’ inhaler and therefore continued using it, understanding that it would continue to keep them well. Previously they would have assumed that they did not need medication when they felt well. One participant reported that they had not linked the terminology of ‘preventer’ with the fact that the medication was designed to prevent an asthma event from occurring.

The overall results from the study confirmed the research question that a nurse‐led education programme can promote compliance with inhaler use in patients with asthma. This study has shown that inhaler technique education has improved participant inhaler technique, participant confidence levels in relation to self‐administration of their inhaler and participant adherence to prescribed frequency of use. As well as nurses, the findings of the study have implications for other professionals including medical doctors and pharmacists. It is suggested that when medicine is being prescribed, dispensed and administered, professionals have a responsibility to maximize patients’ education and understanding.

### Limitations

The study was completed in the fulfilment of a Masters in Science (Research); this placed time constraints on data collection and hence limited the sample size. It also limited the follow‐up period. It would have been very useful to assess whether or not correct inhaler technique and compliance were sustained over a longer time frame and what impact continued\discontinued education would have on the results. Other limitations include the familiarity that grew between the researcher and the participants over time.

## Conclusions

This study confirms that patients do not use their inhalers to optimal effect. Inhaler education at time of first introduction, with regular reinforcement at points of contact with healthcare professionals, is essential. Patients are not convinced that they need to adhere to inhaler regimens when they are symptom free. Structured evaluation of technique is a priority when reviewing an asthma patient using an inhaler.

This study demonstrates that repeated inhaler technique education seems to improve both intentional and unintentional non‐adherence to inhaled medication regimens in asthma. In this small study, the patients newly prescribed to the Diskus inhaler, who were educated from the outset and regularly over 6 months, maintained good technique. Participants who had previously been prescribed the Diskus, when provided with repeated inhaler technique education, showed significant improvements in technique. All participants showed significant improvement in compliance and adherence.

Participant confidence levels in their inhaler technique were high from the beginning, although with hindsight the participants discovered this confidence was misplaced. With education, they were better able to more correctly state their confidence levels on subsequent visits. The findings of this study and the published literature indicate that there are significant implications for the role of nurses, pharmacists and the Health Service in the delivery of patient education. Nurses have a responsibility to continually question their practice and to provide the highest quality evidence‐based care to their patients.

## Conflict of interest

No conflict of interest has been declared by the authors.

## Author contributions

All authors have agreed on the final version and meet at least one of the following criteria [recommended by the ICMJE (http://www.icmje.org/ethical_1author.html)]:

- substantial contributions to conception and design, acquisition of data, or analysis and interpretation of data;
- drafting the article or revising it critically for important intellectual content.
